# Supplementary material for: The LAMMER Kinase MoKns1 Regulates Growth, Conidiation and Pathogenicity in Magnaporthe oryzae
Source: Int J Mol Sci. 2022 Jul 22;23(15):8104. doi: 10.3390/ijms23158104 (PMC9332457; doi:10.3390/ijms23158104)
Supplement: Supplementary file 1 [file ijms-23-08104-s001.zip › ijms-1740649-supplementary.pdf]

International Journal of
Molecular Sciences

MDPI

Supplementary Materials

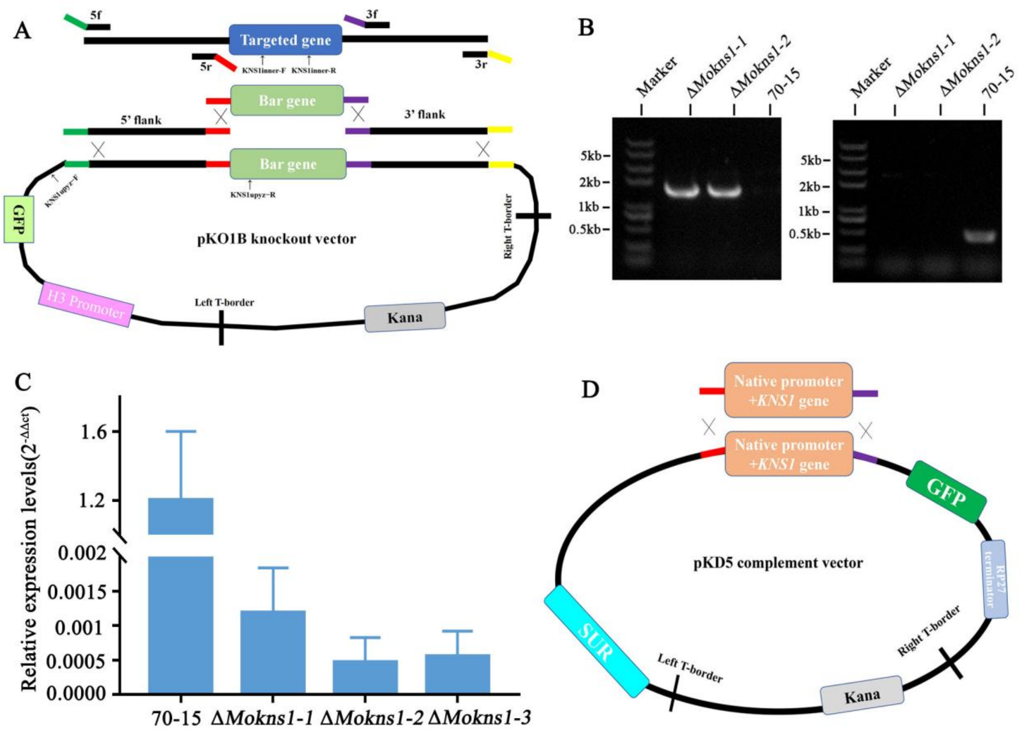

Figure S1. Validation of *MoKNS1* knockout and complemented strains. A. Strategy map for creating deletion Δ*Mokns1* strains. The upstream and downstream fragments of the targeted gene and fragments of the resistance Bar gene were amplified, and the three fragments were introduced into the pKO1B vector by homologous recombination. Kns1inner-F/R primers were designed in the interior of the targeted gene. If the targeted gene was successfully knocked out, the mutant could not amplify this fragment, while the wild-type 70-15 strain could amplify a fragment of about 500 bp. The KNS1upyz-F primer was designed to be about 300 bp upstream of the upstream fragment of the targeted gene, and the KNS1upyz-R primer was designed to be inside the fragment of the resistant Bar gene. For the mutant, it was possible to take advantage of this KNS1upyz-F/R primers, and a band of about 2 kb could be amplified, but the wild-type 70–15 strain could not be amplified. Successful acquisition of knockout mutants could only be demonstrated by using both pairs of primers. B. The targeted gene in the transformant was screened by PCR. A characteristic band (0.5 kb) was amplified from the WT 70-15 strain and the ectopic transformant bu using Primes KNS1inner−F/R, whereas the band was not found in the null Δ*Mokns1* mutant (Right image in Figure B). The specific band (1.5 kb) was amplified from the Δ*Mokns1* mutant bu using Primes KNS1upyz−F/R, whereas the band was not found in the WT 70-15 (Left image in Figure B). The two PCR experiments verified that the targeted gene was successfully replaced by the resistance gene (Bar gene) and the knockout Δ*Mokns1* mutant was obtained. C. The relative expression level of *MoKNS1* gene was verified by qPCR. The expression level of *KNS1* in the three mutant strains was examined, and the expression level in the wild type 70-15 strain was used as a control. D. Strategy map for creating the complementation strains Δ*Mokns1-C.*

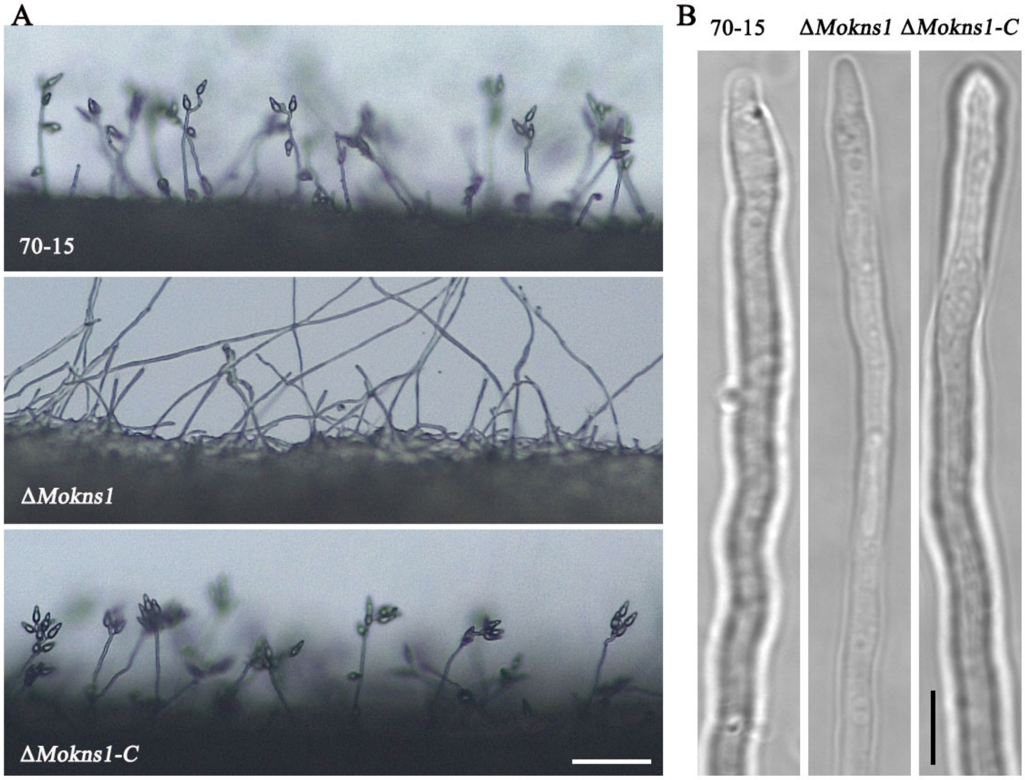

Figure S2. Mycelial and conidiophore morphology. A. Conidiophores morphological differences between the wild-type 70-15 strain and the Δ*Mokns1* mutant were observed using a microscope. Bar, 100 μm. B. Differences in mycelium morphology between the wild-type 70-15 strain and the Δ*Mokns1* mutant were observed under a microscope. Bar, 10 μm.

Table S1. Primers used in this study.

**Name**	**Sequence (5' - 3')**
**Primers used for gene knockout**	
BAR-F	TGCCAACGCCACAGTGCCCCA
BAR-R	TGAGAGCATGCAATTCCCGTG
KNS1up-F	AGGCTAACTGACACTCTAGAACTCTGTTTCGCCGATGGG
KNS1up-R	ACTGTGGCGTTGGCACAAAGGTTGAAGGTGCGGGTT
KNS1dn-F	CTCACGATTCACAATCTCATGGAAGAGGATCAGTATAG
KNS1dn-R	CGACGGCCAGTGCCAAGCTTGTCGCTATGGAGCTTGGTG
KNS1inner-F	ACCAACAGTACCATTCGAC
KNS1inner-R	TGGAGTTGGCGGGTGCACCATT
KNS1upyz-F	GGAGGCAAAATAGAAAAGAGG
KNS1upyz-R	AGGGCGAACTTAAGAAGGTATG
Primers used for yeast two-hybrid assay	
KNS1-N1-AD-F	GGAGGCCAGTGAATTCATGTCGACTCCAACAACTGCA
KNS1-N1-AD-R	CGAGCTCGATGGATCCCTTGTAGAAGTTATCCCAGTC
KNS1-N2-AD-F	GGAGGCCAGTGAATTCTTACAGTCGACCGCCCCCGGT
KNS1-N2-AD-R	CGAGCTCGATGGATCCTCTTCTCTTCGCCTGACCCGC
KNS1-N3-AD-F	GGAGGCCAGTGAATTCTATCAAATGATCAAACTTCTA
KNS1-N3-AD-R	CGAGCTCGATGGATCCAAACCAAGGGTGTTGCAGGGC
ATG18-BD-F	CATGGAGGCCGAATTCATGGCGACTGCAACGCTAAACTT
ATG18-BD-R	GCAGGTCGACGGATCCAGACTCATATGTCGAAGAAGAG
Primers used for complement	
KNS1-GFP-F	ATCACAATGGCCGGATCCATGTCGACTCCAACAACTGCA
KNS1-GFP-R	CTTGCTCACCATCCCGGGATCTTGTCGCTCGGCTTGCCT
Primers used for pull-down	

ATG18-His-F	CAAGGTCGACAAGCTTATGGCGACTGCAACGCTAAAC
ATG18-His-R	GTGCGGCCGCAAGCTTAGACTCATATGTCGAAGAAGA
KNS1-N2-GST-F	CCGCGTGGATCCCCGGAATTCTTACAGTCGACCGCCCCCGGT
KNS1-N2-GST-R	CTCGAGTCGACCCGGGAATTCTCTTCTCTTCGCCTGACCCGC
KNS1-N3-GST-F	CCGCGTGGATCCCCGGAATTCTATCAAATGATCAAACTTCTA
KNS1-N3-GST-R	CTCGAGTCGACCCGGGAATTCAAACCAAGGGTGTTGCAGGGC
Primer used for qPCR	
q-ACTIN-F	ACAATGGTTCGGGTATGTGC
q-ACTIN-R	CGACAATGGACGGGAAGAC
q-CDC15-F	CAGAAAGACCCCAATCTCAGAG
q-CDC15-R	TGTTCCACTGCTTGACCTC
q-CDC14-F	AATGTTCTCTGCCACTTCTCC
q-CDC14-R	CAAGGGACTCGAAATAGGATGG
q-DBF2-F	AAGAGTGAATGGCTGGTTAGG
q-DBF2-R	TGTTAAGGAGTGTGCGGAAG
q-NUD1-F	AGCTGAATGATAAGAGGCTCG
q-NUD1-R	TGATTATGGGCGATCTTGAGG
q-MOB1-F	CGAAAACTTCAAGAGGCCAAC
q-MOB1-R	GCGAACAAGAGATGGGAATG
q-CDC5-F	CAACGGGTCTCTGATGGATATG
q-CDC5-R	TTCCCCATCTTCAAGTCACG
q-TEM1-F	GTATCTGAACCATGACATCCCC
q-TEM1-R	CGGTGGCGTAGGACTTTC
q-RAD17-F	AACCTGAGAAACTATCGCCC
q-RAD17-R	AGAAAGACAGCAAGTACCCG
q-CDS1-F	GACTACGATGAAGACTCCCAAC
q-CDS1-R	GAAAACCCCGAATTCTGCTTG
q-CHK1-F	ACCCTATTCATCTCGCAACG
q-CHK1-R	GTTGTGTAGCAGAGAATTTGGC
q-APC3-F	CACTGGAGAAGCAAAAGCAAG
q-APC3-R	TTTCTGAGCCTCGTGTAGTTG
q-APC1-F	ACCCCTCACCAGACCTATAC
q-APC1-R	TCGGCTGCAAAATTGAAACTC
q-CDC20-F	GAGTCCTCTGGCGTTCTTTC
q-CDC20-R	TGGCGATGGAGTTTTGGTATG
q-MSH2-F	ACAGTCTAGTTCGCTTGCAG
q-MSH2-R	GTCCATCTCAGTCCGTATTCTG
q-RFA2-F	ACAATTTCTCAAGGACGGGC
q-RFA2-R	CACTCGTCTTGGTAGCTCTTG
q-ATG1-F	CAAGTTTACATGGGCAAGCAC
q-ATG1-R	CAATATGAGGGTGTCGGAGAG
q-ATG2-F	ACCATCTATCAACCTCAACCTG
q-ATG2-R	CGGTCGCACAGTAACAATTTG
q-ATG3-F	GAGGATGAAGAGGACGATGAAG
q-ATG3-R	GGACAAGTAGAGACGAGGTG
q-ATG4-F	TTCAATACACAATTTCGTCGCG
q-ATG4-R	GAATTCTCGGTTGCATTGGC
q-ATG5-F	GGGTCTTTTAGGGTCATGCAG
q-ATG5-R	AGCAAGTCACGTAGTGTTGG
q-ATG6-F	GATCTCAACCCAGCCGC
q-ATG6-R	CCTTCCTGTTCTGATCGTGG
q-ATG7-F	CCTCTACCAGCCAATGTCG
q-ATG7-R	AATTCCTGATGCTACCGTCTG
q-ATG8-F	AAGTTCAAGGACGAGCACC
q-ATG8-R	GTCCGACTTTTCTACCTTCTCG
q-ATG9-F	AATGTGGAGATGGGTCAACG
q-ATG9-R	AGGAAGGTTAGGAAAACGGC
q-ATG11-F	CAGGTGTTGATAGCCCACTC
q-ATG11-R	TGATGCAGTTCTGTGGTATCG
q-ATG12-F	ACTCTCAAGGTTGGCAATGG

q-ATG12-R	GCAGGAGTCATGGAGTAAGATAC
q-ATG13-F	GGACTCGGTCAAGAAACTGG
q-ATG13-R	GTATTAAGTCTGGCCTGGATGA
q-ATG14-F	CTGAGGTTGTCGATTTGCATG
q-ATG14-R	GATAGTCAGCGTGTGGAAGG
q-ATG15-F	TGAAAGCAATGGAACGGAGAG
q-ATG15-R	TTCATCCAAGCATCCCCAC
q-ATG16-F	AGTATCTCGCGTCCATCAAAG
q-ATG16-R	TGAGGCTCCTGATGCTTTTAG
q-ATG17-F	AGGAGCCGAGAAGTTTGATG
q-ATG17-R	GCGGATGTCGTTGTCAAAAC
q-ATG18-F	GACTGCAACGCTAAACTTCATC
q-ATG18-R	CGTCTTCGCTCGAGAATATCTTG
q-ATG20-F	GGTTTCTAGATCCGAACGCTAG
q-ATG20-R	GGGAAGATAACTATGGGCAGG
q-ATG22-F	GGAGACGTATGTGATCTGCG
q-ATG22-R	CTGGAGTCTTGTCGTAGCTTG
q-ATG23-F	GCTGGAGAAGGTTCACGAAG
q-ATG23-R	CTGAGGGTCCTTAATGTTCTCG
q-ATG24-F	ATGATAGTTCACGGCATGGG
q-ATG24-R	CGCATCGGGTTTTGAAAGTC
q-ATG26-F	CGCATGATTGTCAAAGCACG
q-ATG26-R	CGGTTGCTGTTTCGTATTCTTG
q-ATG27-F	TGACAAGGACAAGGACAAGG
q-ATG27-R	AGAGCCAAAGATGAGGTATGC
q-ATG28-F	AGAGAAAGGAGACACAACGC
q-ATG28-R	GGGTATCTCATTATCGCTCTCC
q-ATG29-F	AGCTCTGTGGAACATTCTGTC
q-ATG29-R	ATGGCGTTCTGTAAGGTACG
